# Self-organized cell assembly formation

**DOI:** 10.1186/1471-2202-15-S1-P30

**Published:** 2014-07-21

**Authors:** Timo Nachstedt, Florentin Wörgötter, Christian Tetzlaff

**Affiliations:** 1 Third Institute of Physics, Georg-August-Universität, Göttingen, 37077, Germany; 2 Bernstein Center for Computational Neuroscience, Göttingen, 37077, Germany

## 

Cell assemblies are groups of highly interconnected neurons [1]. They emerge due to correlated neuronal activity, for instance induced by external inputs. Correlated activity, in turn, leads to modifications of the synaptic efficacies via synaptic plasticity. Thus, the cell assemblies represent features of the external inputs and are likely to play an important role in the formation of associative memory in the neocortex [2]. Various models have been published demonstrating different ways of formation and functional usage of cell assemblies [3]. However, in most of these models the subset of neurons forming a cell assembly is chosen purely randomly or artificially predefined. A potential solution to overcome this shortcoming lies in the principles of self-organizing maps (SOMs) [4]. In SOMs, the incoming synaptic efficacies are adapted to allocate different input patterns to different groups of neurons. Here, we combine the principles of SOMs as well as those of cell assembly formation to demonstrate the self-organized emergence of highly input specific assemblies.

The neural network used in this study consists of several independent input neurons that project via plastic synapses to a plastic recurrent neural network. Every network neuron receives excitatory synapses from all input neurons. Initially, the efficacies of these synapses are random. Furthermore, each network neuron receives excitatory inputs from other network neurons within a given radius. Inhibition is introduced by a global inhibitory neuron that is mutually interconnected with each network neuron via non-plastic synapses. All neurons are modeled by rate-based leaky integrator units. The projective synapses from the input neurons to the layer neurons, as well as the excitatory synapses in between the layer neurons, are governed by a generic combination of Hebbian plasticity and synaptic scaling [5].

Alternately, different subsets of input neurons are activated for a fixed duration. Comparable to self-organizing maps, the interplay of correlation based strengthening of the projective synapses and synaptic scaling leads to a selective tuning of the neurons to one of the input patterns. The lateral excitatory synapses result in a clustering of similarly tuned neurons. At the same time, the plasticity of the lateral network synapses enhances the efficacies between neurons belonging to the same cluster and depresses efficacies between neurons of different clusters. As a result, the structure of the input clusters is manifested in the efficacies of the lateral connections. Input-specific cell assemblies are formed in a self-organized manner.

We analyze the dynamics of this model, for instance in dependence of the training procedure. For disjunct and temporal sufficiently separated input sets, cell assemblies are learned fast and reliable. However, if in the initial phase two different sets of inputs are activated in quick succession, transient activity in the respective network clusters leads to an initial overlap of these. The system is not able to discriminate them. Only with ongoing experience, the respective clusters start to slowly shift from each other. Similar dynamics occur if the different sets of input neurons overlap. These results indicate an interesting interplay between inputs and the self-organized formation of cell assemblies.
